# Patient and clinician characteristics and preferences for increasing participation in placebo surgery trials: a scoping review of attributes to inform a discrete choice experiment

**DOI:** 10.1186/s13063-022-06277-x

**Published:** 2022-04-12

**Authors:** Madeleine Hinwood, Laura Wall, Danielle Lang, Zsolt J. Balogh, Angela Smith, Michelle Dowsey, Phillip Clarke, Peter Choong, Samantha Bunzli, Francesco Paolucci

**Affiliations:** 1 School of Medicine and Public Health, University of Newcastle, Newcastle, Australia; 2 Hunter Medical Research Institute, New Lambton Heights, Australia; 3 Newcastle Business School, University of Newcastle, Newcastle, Australia; 4 Department of Traumatology, John Hunter Hospital and the University of Newcastle, Newcastle, Australia; 5 Hunter New England Local Health District, Newcastle, Australia; 6 Department of Surgery, St Vincent’s Hospital, University of Melbourne, Australia, Fitzroy, Australia; 7 School of Population and Global Health, University of Melbourne, Australia, Parkville, Australia; 8 Health Economics Research Centre, University of Oxford, Oxford, England

## Abstract

**Background:**

Orthopaedic surgeries include some of the highest volume surgical interventions globally; however, studies have shown that a significant proportion of patients report no clinically meaningful improvement in pain or function after certain procedures. As a result, there is increasing interest in conducting randomised placebo-controlled trials in orthopaedic surgery. However, these frequently fail to reach recruitment targets suggesting a need to improve trial design to encourage participation. The objective of this study was to systematically scope the available evidence on patient and clinician values and preferences which may influence the decision to participate in placebo surgery trial.

**Methods:**

A systematic review was conducted via a literature search in the MEDLINE, Embase, PsycInfo, CINAHL, and EconLit databases as of 19 July 2021, for studies of any design (except commentaries or opinion pieces) based on two key concepts: patient and clinician characteristics, values and preferences, and placebo surgery trials.

**Results:**

Of 3424 initial articles, we retained 18 eligible studies. Characteristics, preferences, values, and attitudes of patients (including levels of pain/function, risk/benefit perception, and altruism) and of clinicians (including concerns regarding patient deception associated with placebo, and experience/training in research) influenced their decisions to participate in placebo-controlled trials. Furthermore, some aspects of trial design, including randomisation procedures, availability of the procedure outside of the trial, and the information and consent procedures used, also influenced decisions to participate.

**Conclusion:**

Participant recruitment is a significant challenge in placebo surgery trials, and individual decisions to participate appear to be sensitive to preferences around treatment. Understanding and quantifying the role patient and clinician preferences may play in surgical trials may contribute to the optimisation of the design and implementation of clinical trials in surgery.

**Supplementary Information:**

The online version contains supplementary material available at 10.1186/s13063-022-06277-x.

## Introduction

Historically, new surgical procedures have been introduced based on practical experience [1]. However, there is increasing interest in ensuring that interventional procedures have been validated using RCTs, with the gold standard being a placebo-controlled RCT [2, 3]. This is of particular relevance to orthopaedic surgery, where studies have shown that a significant proportion of patients report dissatisfaction and no clinical meaningful improvement in pain or function after certain orthopaedic surgical procedures [4–7]. Globally, orthopaedic surgery includes some of the highest volume surgical interventions, involving high financial cost and increasing demand. Therefore, it is critical to ensure that these procedures target those who will gain a clinically important improvement from the surgery. Presently, there are large knowledge gaps around which patients are likely to derive benefits from elective orthopaedic procedures. As such, there is growing momentum and support for RCTs to be run in orthopaedic surgery to resolve these issues and evidence gaps.

Testing whether a surgical procedure is superior to a placebo, or sham surgery procedure, is necessary for ensuring efficacy, since results may be compromised by a placebo response [8, 9]. The placebo effect associated with surgical procedures has been found to be larger than seen in non-invasive interventions, particularly for subjective outcomes such as pain and function [10], which is the primary purpose of undergoing the surgery. It is therefore important that placebo effects are considered when interpreting the results of surgical procedures seeking to improve quality of life (as opposed to preserve life). A comparison against placebo surgery (involving anaesthesia and a skin incision to mimic key aspects of surgery) enables researchers to determine whether the interventional element of the procedure has a benefit beyond a placebo effect [11]. Whilst placebo-controlled surgical trials in orthopaedic surgery are increasing in number, many procedures are yet to be evaluated using this framework. As such it has been argued that conclusions regarding the efficacy of some of the highest volume and complex medical interventions remain constrained by significant methodological limitations [12].

Low recruitment is an important methodological barrier to the successful completion of placebo-controlled trials [3, 13]. Patient and surgeon preferences surrounding decisions to participate in placebo surgery trials have a clear impact on the recruitment rate and may also affect adherence to allocated treatments and follow-up, and outcome assessment [14]. Patient and surgeon preferences can thus influence the overall success and validity of these trials.

To ensure the highest quality of evidence can be produced from RCTs in orthopaedic surgery and that research funds, as well as patient and surgeon time, are not wasted, it is important to understand the barriers and enablers to participation. We hypothesise that the decision to participate in a placebo surgery trial is a preference-sensitive decision, and in order to improve trial participation, it is critical to understand the range of patient and surgeon attitudes, values, and preferences around the decision to participate. To more thoroughly explore the feasibility of placebo surgery trials, a systematic review has also been conducted by members of this research team. It investigated the disparities between planned (protocol and trial registry) and actual (outcome papers) outcomes from placebo surgery trials [13]. Whilst the systematic review quantified the extent to which surgical RCTs achieve their recruitment targets, this scoping review will identify published information around patient and surgeon preferences for trial participation.

## Aims and objectives

The objective of this study was to systematically scope the available evidence on themes, including patient and clinician values and preferences influencing the decision to participate in placebo surgery trials. We used a scoping review methodology to map the depth and the breadth of literature in this area, as well as to synthesise what we predicted would be many narrative themes. In addition, this work will be used alongside a qualitative study inform the attributes and levels of a discrete choice experiment (DCE), a quantitative research method commonly used in psychology and health economics [15] to elicit individuals’ preferences by presenting a series of pairs of hypothetical options where attributes thought important for decision-making are varied [16]. This allows for the identification and ranking of the most relevant attributes without explicitly asking participants, the findings of which can then be applied in future settings. For example, in the proposed study, a DCE identifying preferred features of surgical trials will contribute towards the design of more patient-centred clinical trials. The utility of a DCE is reliant upon identifying the attributes which are salient to the scenario being investigated, and the levels over which they vary. Therefore, the choice of attributes should be informed by a range of sources, such as literature reviews and qualitative studies [17–20]. Broadly, the objective of this study is to identify potential attributes via a systematic review of the literature reporting themes associated with recruitment to placebo surgical trials. This scoping review will inform an important stage in the development of a DCE, which will quantitatively estimate the influence of various factors on trial participation that may improve recruitment rates to surgical RCTs.

The rationale for the DCE has been previously described [21]; briefly, the DCE will empirically test patient and surgeon preferences for different placebo surgery trial designs. There is an identified need to improve trial design and increase recruitment in orthopaedic surgery trials, and a well-designed and well-conducted DCE will provide translatable, quantitative information on the relative importance of identified attributes in influencing decisions to participate. Further, in addition to informing the development of a DCE, data on themes associated with participation in placebo trials will be valuable for informing decision-making, patient-centred outcomes research, and future trial design.

## Methods

### Study design and registration

The methodology employed in this review was guided by Arksey and O’Malley’s framework [22], and the Joanna Briggs Institute methodology for scoping reviews [23]. An a priori study protocol was prepared in accordance with the Preferred Reporting Items for Systematic Reviews and Meta-Analyses Extension for Scoping Reviews (PRISMA-ScR) and was published in 2020 [21]. This review was reported according to the PRISMA Extension for Scoping Reviews (PRISMA-ScR; Additional file 1: Appendix 1) [24].

The primary questions that guided this review were:

- What are patients’ characteristics, values, and preferences around choosing to participate in a placebo surgery trial?
- What are clinicians’ (surgeon and anaesthetists) characteristics, values, and preferences around choosing to participate in a placebo surgery trial?
- What are the trial characteristics that may influence patient and clinician decision-making around placebo surgery trials?
- How might these themes contribute to a DCE as attributes and levels?

There was one major deviation from the originally planned protocol, that is the use of the Mixed Methods Appraisal Tool (MMAT) [25] to assess study quality, rather than the Cochrane collaboration’s tool for assessing the risk of bias in RCTs [26] or the consolidated criteria for reporting qualitative studies [27], as it allowed for the concomitant evaluation of the range of study designs identified in the search.

### Data sources and search strategy

In order to determine themes around participation in placebo-controlled surgical trials, we conducted a systematic search of published studies describing patient and clinician preferences via any method to inform this scoping review.

We conducted a systematic search of the literature following the Cochrane Collaboration guidelines and reported our findings using the PRISMA-ScR [24]. Our search criteria were informed by the two key concepts of placebo surgery trials, and decision-making around participation. In the search strategy, we used terms for both sham and placebo to describe the placebo control. Both are used in the literature to describe surgical placebos, which aim to imitate the investigated intervention. We included all placebo surgery trials in our literature search as data for orthopaedic placebo surgery trials alone were likely to be limited, and patient and surgeon preferences for all placebo surgery trials are likely to be relevant.

The search strategy was developed in consultation with a senior research librarian in MEDLINE before being translated to the other databases. The following databases were searched from database inception using medical subject headings and keywords: MEDLINE (OVID; indexed since 1966), Embase (OVID; indexed since 1974), PsycINFO (OVID; indexed since 1967), EconLit (EBSCOhost; indexed from 1886), and the Cumulative Index to Nursing and Allied Health Literature (CINAHL [EBSCOhost]; indexed since 1984), using text and MeSH terms exploded to include all subheadings. The literature search was conducted on 24 October 2019 and updated on 19 July 2021. The search strategy is outlined in Additional file 2: Appendix 2.

### Study selection

Search results were imported into Endnote X8 (Clarivate Analytics), and duplicates were removed. Three authors conducted title and abstract screening (LW, DL, MH). Each title and abstract were screened by two authors, and conflicts were resolved by consensus with a third author. Full texts of potentially relevant records were retrieved and assessed for eligibility in Covidence referencing software (Veritas Health Innovation). Reference lists of all potentially eligible reviews were also searched for additional titles relevant to the search, as well as to search for methodological details missing from the included report, as necessary. All published manuscripts from any year or country were included. Publications were limited to studies conducted in humans and published in the English language

Any empirical studies or reviews that provided qualitative or quantitative data on themes related to participation in placebo surgery trials were included, such as focus groups, interviews, surveys, clinical studies/RCTs, and reviews. Abstracts, books or book chapters, editorials, letters, and notes were excluded. The eligibility criteria are described below according to the PEO (Population, Exposure, Outcomes or themes) variation on the PICO (Population, Intervention, Comparator, Outcome) framework for qualitative approaches [28]:

*Population*: The population of interest was patients, clinicians, or other relevant stakeholders involved in, or considering involvement in, a sham or placebo surgery trial in any setting.

*Exposure*: A placebo surgery trial in any therapeutic area, defined as a trial in which a surgical technique is compared with a blinded procedure intended to emulate that surgery (such as anaesthesia and a skin incision). Ineligible study designs included trials assessing a procedure for drug delivery comparing the procedure with and without the drug, as opposed to the procedure (e.g. drug-eluting stent), or a procedure that was not considered surgery (e.g. nerve stimulation for the treatment of migraine).

*Themes and outcomes*: Any studies that evaluated themes including values, attitudes, beliefs/understanding and preferences of patients and/or clinicians, or trial and recruitment characteristics, that affect participation in placebo surgery trials were included. Outcomes could have been reported as a qualitative synthesis of interviews or focus group data, through quantitative measures, or in a narrative review.

### Data charting and synthesis of results

Data were extracted from eligible studies by three of the authors (LW, MH, DL) and charted using a standardised data abstraction form developed in Excel (Microsoft). The form was developed by the authors for the study and was designed to capture article details as well as any relevant themes around participation in surgery trials. The form was piloted on four studies, completed by all three authors, in order to ensure it captured the required data sufficiently. The following information was extracted for each study: bibliographic details, study type, description of dataset used; and relevant themes associated with decisions to participate in placebo surgery trials, categorised by patient, clinician (e.g. surgeon or anaesthetist), and trial design characteristics. The latter were extracted for thematic analysis of factors associated with recruitment to placebo surgical trials. The data extraction form is provided in the supplementary Appendix.

Results were analysed using inductive thematic synthesis and summarised narratively. The findings were broadly classified within the three categories used to guide data extraction; that is, either patient characteristics, preferences or values; surgeon characteristics, preferences, or values; and trial or consent procedure characteristics influencing participation in placebo surgery trials. Individual themes were identified inductively after completion of the systematic search and data extraction. These findings were reframed as possible DCE attributes and levels, grouped where appropriate, and summarised in a table. Levels were assigned to the broader themes or conceptual attributes, where appropriate; these were qualitatively identified based on information in the sources and included domains by which the identified themes could conceivably vary over.

### Critical appraisal of individual studies

In line with the recommendation made by Arksey and O’Malley [22], that quality assessment does not form part of the scoping review remit, eligible studies were included in the review regardless of their methodological quality or risk of bias. However, a quality assessment was conducted to gain an understanding of the methodological quality of the available evidence. For quantitative, qualitative, or mixed-methods studies utilising surveys, interviews, focus groups, or DCEs, we evaluated study quality using the Mixed Methods Appraisal Tool (MMAT), version 2018 [25, 29]. Papers were assessed independently by two reviewers (out of LW, DL, and MH) for methodological validity. The MMAT was chosen as an appraisal tool in this study due to the range of methodological designs identified by the search; it allows for the concomitant evaluation of empirical qualitative, quantitative, and mixed methods studies. The criteria, from Hong et al. (2018 [25];) are included in Additional file 4: Appendix 4; briefly, all included studies are subjected to the same two screening questions (“Are there clear research questions”; “Do the collected data allow to address the research questions?”), and a follow-up appraisal with questions dependent upon study type. The MMAT cannot be used for non-empirical papers including narrative review or theoretical papers. There is a lack of standard approach to assessing the quality of normative papers such as these, and as such, a pragmatic approach to quality assessment was adopted in this review, whereby the quality of these papers was considered sufficient if they appeared in an international peer-reviewed journal. This approach to quality assessment has been used in other reviews [30, 31].

## Results

**Fig. 1:**
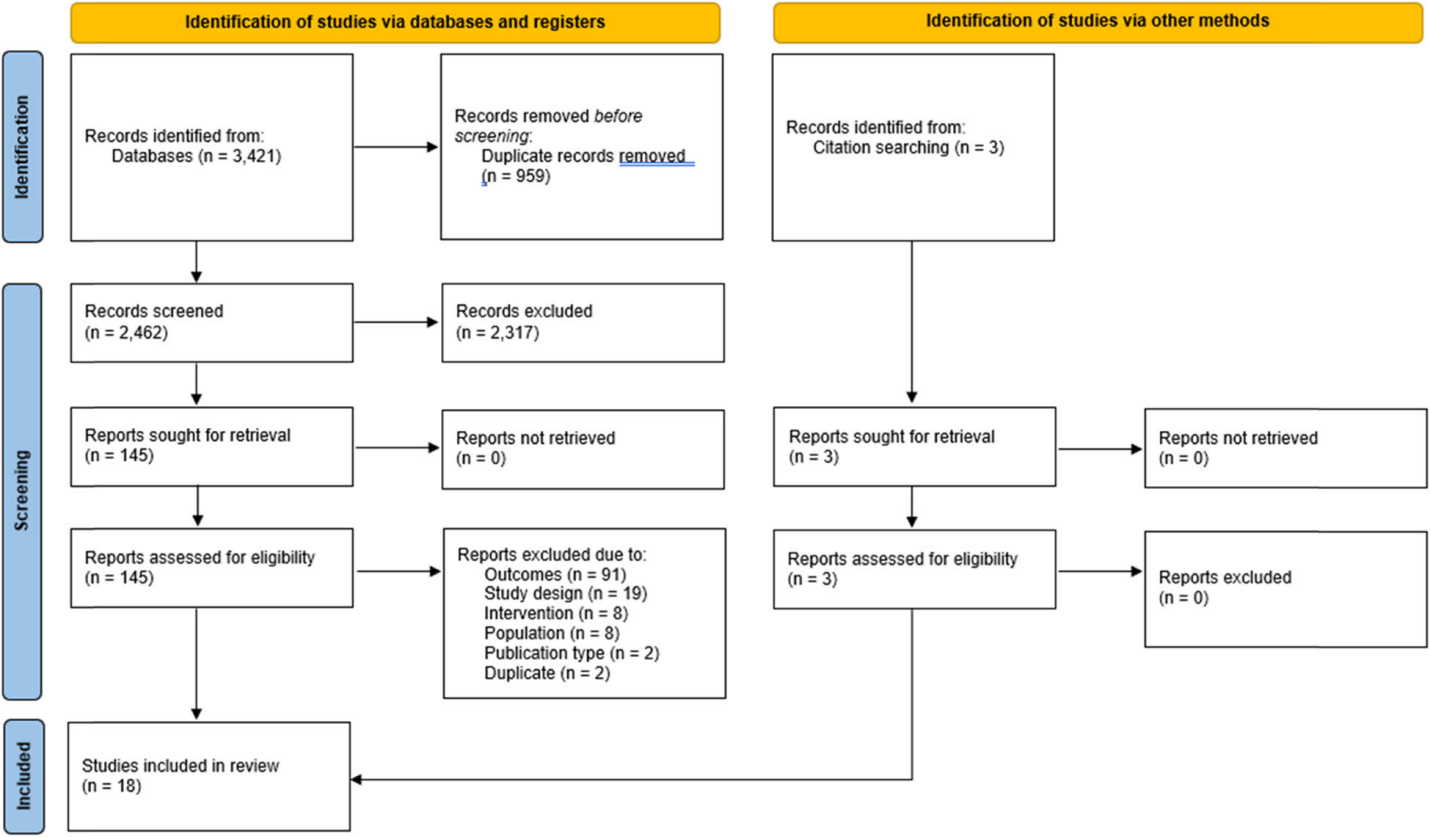
PRISMA flow diagram [32]

Overall, the search strategy identified 2462 titles (after 959 duplicates were excluded). Based on the titles and abstracts, 145 papers were ordered and manually reviewed. Hand searching of reference lists resulted in full-text screening of another 3 papers. Of these 148, 18 were included in this study (Fig. 1).

Included studies are summarised in Table 1. The included studies were conducted in primarily high income countries, and comprised a variety of publication types, incorporating survey and questionnaire studies, [12, 33–35, 39]; interviews and focus groups [36, 37, 42–44, 46]; participation interviews or other follow up attached to randomised controlled trials [40, 41, 45]; narrative reviews [38, 48]; and guidelines for the conduct of trials [47, 49].

**Table 1:** Characteristics of included studies

Bibliographic information	Country	Methodology	Surgical speciality/field	Participants/included studies
Anderson et al. (2019) [33]	Australia	Semi-structured interviews	Lumbar decompression surgery	*N*=63 People diagnosed with central lumbar spinal canal stenosis considered suitable for lumbar decompression surgery
Baldwin, Wartolowska and Carr (2016) [34]	UK	Online survey	Orthopaedic surgery	*N*= 189 Members of the British orthopaedic trainees association
Campbell et al. (2010a) [35]	USA	Narrative review and case study	Orthopaedic/general surgery	Surgical RCTs and a case study of a paediatric orthopaedic RCT
Campbell et al. (2010b [36]; 2011 [37])	UK	Mixed methods/feasibility study (surveys, interviews, and focus groups)	Orthopaedic surgery	*N*=41 surgeons (three focus groups); *N*=130 orthopaedic anaesthetists (plenary discussion); *N*=58 anaesthetists (three focus groups); *N*=7 members of patient organisation Arthritis care (two focus groups); *N*=15 people on consultant waiting lists for treatment (telephone interviews); *N*=6 chairs of UK ethics committees (interviews); *N*=382 members of the British Association of Surgeons of the Knee (postal surveys); *N*=398 members of the British Society of Orthopaedic Anaesthetists (postal survey) *N*=49 patients (two centre pilot study)
Cook et al. (2009) [38]	UK	Narrative review	General surgery	Clinical trials conducted in surgery
Frank et al. (2008) [39]	USA	Survey study	Neurosurgery (gene therapy via intracerebral delivery)	*N*=56 people with Parkinson’s disease; *N*=113 non-PD neurology patients; *N*=119 primary care patients
Hare et al. (2014) [40]	Denmark	Randomised controlled trial (participation interview)	Orthopaedic surgery	*N*=40 people with referrals for suspected medial meniscus lesion
Kim et al. (2012a) [41]	USA	Semi-structured interviews	Neurosurgery (cellular and gene transfer)	*N*=90 people who were participating in one of 3 sham-controlled intervention trials for Parkinson’s disease
Kim et al. (2012b) [42]	USA	Semi-structured interviews	Neurosurgery (cellular and gene transfer)	*N*=71 61 enrolees and 10 decliners of participation in 2 invasive placebo surgery controlled randomised trials.
Kim et al. (2013) [43]	USA	Semi-structured interview	Neurosurgery (gene therapy via intracerebral delivery)	*N*=29 People considering enrolment in an early-phase gene transfer trial for Parkinson’s disease
Kim et al. (2015) [44]	USA	Semi-structured interviews	Neurosurgery (cellular and gene transfer)	*N*=90 people with advanced Parkinson’s disease enrolled or intending to enrol in one of 3 placebo-controlled neurosurgical trials
Rios et al. (2021) [45]	USA	Summary and recommendations from a prematurely closed randomised placebo-controlled trial	Paediatric orthopaedic surgery	Discussion of a randomised, placebo-controlled trial of bone morphogenetic protein graft at the time of tibial surgery
Swift (2012) [46]	UK	Semi-structured interviews	Gene therapy via intracerebral delivery	*N*=20 People with Parkinson’s disease considering enrolment in an early-phase gene transfer trial for Parkinson’s disease, and their family members, friends, or carers
Tuszynski et al. (2007) [47]	USA	Guidelines for the conduct of spinal cord injury trials	Surgical interventions for spinal injury	Surgery trials for interventions including including decompression, targeted drug delivery, and spinal laminectomy
Wartolowska, Beard and Carr (2014) [12]	UK	Online survey	Orthopaedic surgery	*N*=100 Orthopaedic shoulder surgeons
Wiebe (2003) [48]	UK	Narrative review	Neurosurgery	RCTs of surgical interventions for epilepsy
Wright et al. (2011) [49]	USA and Canada	Outcomes of the Clinical Trials in Orthopaedics Research Symposium	Orthopaedic surgery	RCTs in orthopaedics

Sampled populations included surgical patients, surgeons and/or anaesthetists. One study included a sample ethics committee chairs alongside patients and clinicians [37]. Most studies evaluated preferences around participation in placebo surgery trials from the patient’s point of view (14 studies; 78% [33, 35, 37–48]). Surgeon and anaesthetist preferences for enrolling patients in placebo surgery trials were considered in 7 (39%) studies [12, 34, 35, 37, 47–49]. While the studies involving surgeons were all in orthopaedics, the studies which included patients covered a number of conditions (lumbar spinal canal stenosis [33], meniscal lesion [40], osteoarthritis [37], and Parkinson’s disease [36, 39, 44, 46]). Patients were also recruited from a number of different stages of trial enrolment including patients eligible for a surgical procedure or recruited from a clinic [33, 37, 39, 46], patients who had agreed or expressed a wish to participate in an RCT [40] and patients who refused to participate in an RCT [42].

### Patient characteristics, preferences, or values influencing decisions to participate

Patient themes that were associated with the decision to participate in a placebo surgery trial are summarised in Table 2. One of the primary aims of this review was to inform a survey and DCE which quantitatively measures patient and clinician preferences. To assist with this aim, the following tables include a column that provides the potential levels of each attribute where applicable, for inclusion in a DCE, or as response options in a multiple choice question as part of the broader survey instrument.

**Table 2:** Patient characteristics, preferences, or values which influenced decisions to participate in placebo-controlled surgical trials

Potential attribute	Potential levels	References
**Demographics**		
Gender	Male Female Other	[41]
Education	Did not finish high school High school/equivalent University level	[33, 39, 42]
**Disease characteristics**		
Level of disability	No impact on functional activities Some impact on functional activities Severe impact on functional activities	[33]
Level of pain/functional status	No pain/functional impairment Some pain/functional impairment Severe pain/functional impairment	[33]
Stage of disease	Early (< 6 months) Medium term (6–24 months) Long term (> 24 months)	[46]
Perception of requiring immediate treatment (e.g. due to work or age)	Believe I require immediate treatment Do not believe I require immediate treatment	[33]
**Individual characteristics**		
Perceptions of risk (associated with experiment/surgery)	Low High	[33, 39, 41, 42, 44, 46]
Individual tolerance of risk/risk appetite	Low High	[42, 46]
Altruism (as an individual characteristic)/able to see benefits to society	Altruistic Not altruistic	[37, 39, 40, 42, 44, 47]
**Trial preferences**		
Perceived therapeutic benefit associated with trial participation	Therapeutic benefit No therapeutic benefit	[42, 43, 46]
Preference for a treatment arm (e.g. due to concerns about the effectiveness or adverse events)	Placebo arm Active treatment arm	[33, 35, 38, 41, 45, 46, 48]

The level of education of potential participants was associated with their willingness to approve of the use of placebo surgery controls in several studies, although with conflicting results. In one study, participants with a college level of education were more likely to agree with the use of placebo surgery controls under hypothetical circumstances, compared with participants who had a high school education or less [39]. Conversely, another study found that participants with a higher level of education were less likely to enrol in a placebo surgery trial [42], whilst another study reported no effect of education [33].

Certain clinical characteristics of patients were associated with decisions to participate in surgical trials. In general, patients with greater levels pain and poorer function were less likely to participate, citing an unwillingness to risk receiving placebo rather than the active arm [33]. Patients with Parkinson’s disease however were more likely to participate in a placebo surgery trial if their disease was at a more advanced state [46]. Patients who required immediate treatment due to other characteristics (such as age, or work; unrelated to clinical characteristics such as level of pain) were less likely to participate [33].

Personal characteristics, particularly risk-benefit perception and altruism also emerged as frequent themes influencing decisions to participate. Individual perception of risk influenced decisions to participate in surgical trials, with people who perceived trial participation to involve a high level of risk less likely to agree to participate [33, 39, 41, 42, 44, 46]. Similarly, people who declined to participate in a placebo surgery trial cited the potential harms as one of the factors influencing their decision, and perceived a higher risk of harm with the trials compared with those who enrolled [42]. In one interview study, participants consciously attributed their hypothetical participation decision to their own personal tendencies toward or against risk-taking in general [46]. Interestingly, some patients felt that the risk: benefit ratio of placebo surgery was positive (i.e. would involve a lower risk of adverse events, but include the benefits of any placebo effects), and would choose to participate because of the chance of being assigned to this [46]. However, overall, people who enrolled in placebo surgery trials also did so with a desire for some therapeutic benefit to themselves, primarily hoping to be enrolled in the active treatment arm [42–44]. Strong preferences for one treatment arm, and the resulting dislike of randomisation requirements of an RCT are often reasons for refusal to participate [33, 36, 38, 41, 45, 46].

Although participants who agreed to participate in a placebo surgery trial perceived the risk of the trial to be lower, compared to people who declined to participate, they were also willing to tolerate a higher level of risk and a lower benefit to self, compared to those not willing to participate [42]. Patients who agreed to participate in placebo surgical trials also frequently expressed more explicit altruistic tendencies, such as citing a desire to participate for the good of society or to help progress research [37, 39, 40, 42, 44, 47]. People who enrolled in placebo surgery trials would participate when there was a high chance of societal benefit, even if there was no chance of personal benefit, whereas the majority of decliners said they would not participate when there was no chance of direct personal benefit, even if there were a 100% chance of societal benefit [42]. The information around possible benefits was also important; patients cited a need for reassurance about positive outcomes after placebo surgery, and willingness to participate increased if provided with more certainty about a good outcome following participation in the trial [33].

### Clinician characteristics, preferences, or values influencing decisions to participate

A summary of themes that may influence clinician (primarily surgeons and/or anaesthetists) decisions to participate in placebo surgery trials, or refer patients to trials, is presented in Table 3.

**Table 3:** Clinician demographic, clinical, and dispositional characteristics influencing decisions to participate in placebo surgery trials

Attribute	Potential levels	References
Concerns around deceiving patients	Concerns around deceiving patients outweigh potential benefits of trial Not concerned/concerns outweighed by the benefit of conducting trial	[12, 34, 48]
Informed consent (difficulties)	N/A	[35, 47, 48]
Potential violation of trust in patient-doctor relationship	N/A	[12, 34, 48]
Legal implications	N/A	[12, 34]
Level of invasiveness of sham/placebo surgery	Anaesthetic (sedation versus general) Size of incision Manipulation beyond incision	[34, 37]
Perceived adverse events/risks associated with placebo surgery	High likelihood of adverse events Low likelihood of adverse events	[12, 37]
Additional burden on surgeons/hospitals associated with trial involvement	Additional administrative burden No additional administrative burden	[49]
Funding arrangements. Trials may be underfunded leaving some costs to be borne by hospitals, insurers, or individuals	Trial is fully funded (including all surgery costs and surgeon time) Trial is underfunded (e.g. some trial costs being met by hospital or insurer budgets)	[37, 49]
Dislike of uncertainty	Comfortable with uncertainty Uncomfortable with uncertainty	[48, 49]
Observations/understanding of placebo components of existing surgical procedures	Believe that some surgical procedures have placebo components/effects Do not believe that some surgical procedures have placebo components/effects	[34, 37]
Training/familiarity with research and clinical trials	Trained and familiar with research Not trained/familiar with research	[49]
Belief in the scientific importance of placebo trials	Believe this level of evidence should be collected for surgical procedures Do not believe this level of evidence is required for surgical procedures	[34]

Clinician concerns around deception or trust were frequently reported, with surgeons unwilling to participate in the blinding associated with placebo surgery trials or concerned about violating the trust in the surgeon-patient relationship [12, 34, 48]. As the surgeon performing the procedure cannot be blinded to randomisation, they may feel as though they are deliberately deceiving the patient to maintain the blinding, which may extend throughout follow-up periods [12, 34]. Potential legal implications were also cited as a frequent concern [12, 34], as was difficulty in obtaining informed consent [35, 47, 48]. Surgeons also expressed a dislike for discussions involving uncertainty for patients, particularly in terms of blinding, and some cited feeling personally responsible if the randomised treatments were found to be unequal [48, 49]. Surgeons with a strong belief that one treatment arm was truly superior to the other may also refuse to participate [35, 48].

Surgeons also had concerns regarding the trial and placebo procedures themselves. Clinicians reported being apprehensive about the potential side effects associated with the placebo procedure and anaesthetic [33, 37], as well as practical difficulties involved in undertaking trial procedures (e.g. extra time taken to explain trial procedures, additional appointments required) [48].

Experience or training in research also influenced decisions to participate. Although the perceived conflict between roles as a scientist and a clinician led to an increased likelihood of declining to participate [48], greater prior experience with research methods or training in clinical trials was associated with an increased willingness to participate [34, 49]. A belief in the scientific importance of the use of placebos to assess the effectiveness of interventions was also associated with an increased willingness to participate [34]. Similarly, surgeons who used procedures which they believed had a significant placebo component were also more likely to be willing to recruit patients to a surgical trial [34, 37].

### Trial characteristics and information and consent procedures

Certain study design features were also more likely to be associated with recruitment rates. These fell broadly into two categories; those related to the procedures and study design (trial characteristics), and those related to the information provided to participants (information and consent procedures). Where the level or type of information provided about particular topics, or comments surrounding the method or content of consent processes was identified as influential, these were classified as *information and consent procedures*. Generally, these characteristics affected patient decisions to participate (e.g. by ensuring patient-centred design or improving understanding and access to Information); however, some characteristics also influenced clinician decisions to participate (e.g. collaboration with other clinicians). Relevant trial and information characteristics are summarised in Table 4.

**Table 4:** Trial characteristics and information/consent procedures associated with differences in recruitment rates

Potential attribute	Potential levels	References
**Trial characteristics**		
Involvement of other clinicians	Surgeons only are involved in trial Surgeons and physicians (e.g. neurosurgeon and neurologist) work collaboratively on trial	[45]
Patient involvement in the trial design	Trial has been designed based on patient perspectives Trial has not had patient input into the design	[45]
If usual clinician is involved in trial	Usual clinician involved in trial Different clinician involved in trial	[44]
Study arm allocation	Random allocation to study arm Patient preference for study arm informs allocation	[33, 35, 39, 45]
Number and type of arms	Two – intervention and placebo Three – intervention and placebo and treatment as usual/non-surgical (e.g. physiotherapy) Three – intervention, placebo one [incision only], and placebo two [more invasive placebo surgery]	[34]
Randomisation ratio	1:1 placebo and active intervention 1:2 placebo and active intervention	[37]
Invasiveness of procedure	Invasive procedure (e.g. joint replacement) Less invasive procedure (e.g. arthroscopy)	[34]
Invasiveness of placebo arm (including anaesthetic use)	Use of local/regional (with sedation) or general anaesthetic; and size of placebo incision (1-10 cm)	[34, 37]
Novelty of procedure (including availability outside the trial)	Procedure available outside of the trial Procedure not available outside of the trial (novel)	[38, 48]
Crossover (availability of procedure if initially assigned to placebo)	Crossover not offered Crossover offered if treatment shown to be effective Crossover offered if desired by patient/ no improvement in symptoms	[41, 47]
Time to follow up and/or crossover	3 months 6 months 12 months	[33, 46, 48]
Financial costs of placebo procedure	Some cost to patient or health system No cost to patient/health system	[37]
**Information and consent procedures**		
Information about benefits (and likelihood of them)	No benefit to self – benefit to future patients and research/society Possible benefit due to placebo effect – and benefit to future people/research/society Possible benefit to you because of extra care during study visits	[33, 35, 44, 47]
Patient understanding about procedure and/or study details	Information about the randomisation process, the study arm(s), and their risks will be included in a standard information sheet for patients to read prior to consenting, with patient understanding not checked prior to consent.Information about the randomisation process, the study arm(s), and their risks will be included in an information sheet for patients to read, *and then their understanding will be checked to ensure it is sufficient*, prior to consenting.	[33, 41, 44, 47, 48]
Definition/terminology of placebo provided to patient	Use of sham vs placebo Standard definition of placebo provided to patient Placebo defined in patient-centred terms, with opportunity for questions	[36]
Comprehensive provision of information about all possible outcomes (including risks)	Patients are informed of possible outcomes and risks through standard discussion with surgeon prior to surgery Patients are informed of possible outcomes and risks through a discussion with a research coordinator in addition to the standard discussion with surgeon prior to surgery	[39, 41, 46, 47]
Patient involvement in the *consent* process (e.g. writing consent in own hand, pre-agreed discussions, formal decision aids)	Standard information and consent form only Information and consent forms accompanied by patient-centred discussion & formal decision aids	[36]
Surgeon involvement in the consent process (and format)	Surgeon involved in consent procedure Surgeon not involved in consent procedure	[40]

#### Trial characteristics

The involvement in the trial of both surgeons (beyond conducting the procedure under investigation) and other clinicians may imply endorsement of the trial to the patient. In trials where surgeons or a patient’s usual clinician were involved in the trial and/or in follow up care, recruitment rates were higher [44]. Greater involvement of multiple clinicians (surgeons and physicians) could also lead to greater recruitment through access to more patients and an overall improved awareness of the trial/condition [45]. Similarly, the involvement of patients in the trial design could lead to improved recruitment through a more patient preferred trial design [45].

A consistent theme across multiple publications was that the presence of randomisation and blinding in a surgical trial posed a threat to recruitment, primarily due to patient discomfort with these concepts [33, 35, 39]. Some authors discuss hypothetical improvements to consent rates for a clinical trial with patient preference trials, where the clinician presents all the treatment arms to the patient and the patient selects the arm they prefer [39, 48]. In another study which summarised responses to open-ended questions, it was found that for 12% of respondents, ‘free choice of which treatment received’ was a factor that would ‘increase willingness to participate in a placebo-controlled trial’ [33]. It is unclear from this study whether that free choice related to a patient preference trial, or a crossover design, and how many participants, if any, would choose the placebo arm. Similarly, in a survey of neurology and general practice patients, regarding a gene therapy trial for Parkinson’s disease (PD), it was found that support for an open, unblinded study was higher than for a blinded, placebo surgery study, such that 83% of patients would `definitely or probably allow’ the open trial but only 54% said the same for the placebo trial [39].

Offering crossover from placebo into the active arm, and the length of time to crossover, were also frequently mentioned as a trial design issue likely to impact decisions to participate. In a qualitative interview study with people eligible to participate in placebo surgery trials for PD, those who would enrol stated that offering the study intervention to those allocated to the placebo arm, at the end of the study, had a strong impact on their decision to participate [41]. The length of time to receive the active treatment in a crossover trial after initially being allocated to placebo was also found to impact the decision to participate in a trial [33, 46].

The invasiveness of both the surgical procedure under investigation, and the placebo procedure used, have also been reported to affect participation [34]. Orthopaedic surgeons have been found to prefer trials investigating a less invasive arthroscopy over open surgery [34]. Similarly, more surgeons would be willing to recruit into a trial where the placebo procedure involved more minimal incisions, sufficient to imitate arthroscopic surgery, compared to a procedure with more invasive skin incisions, enabling exposure and inspection of the joint, sufficient to imitate open surgery [34, 37]. The novelty of the procedure being trialled was also an important factor influencing participation. When a trial is testing a procedure that is novel, and therefore not readily available outside a trial, such as gene or cell therapy in PD, patients tended to be more willing to join a placebo surgery trial [48]. On the other hand, if a procedure is established and easily accessible outside of the trial, particularly if it is perceived as a standard therapeutic option, both patients and physicians were less likely participate in a trial [38, 48].

Perceived and actual costs associated with participation, either to the patient, clinician, or the health system, also emerged as another attribute that may influence participation. Where research funding fails to cover the entire cost of a trial, the direct costs of some included procedures may be borne by the health system, including surgeon and anaesthetist time [49]. Clinicians have expressed concerns about the health system covering some of the costs and resource use of the placebo procedure, including indemnity arrangements, for no expected therapeutic benefit [37]. These factors could potentially affect surgeons’ willingness to be involved and to perform placebo procedures if the costs to themselves and the health system are not completely covered by trial funding. Cost considerations were also found important for a significant proportion of patients; 11.6% of surveyed patients diagnosed with central lumbar spinal canal stenosis suitable for lumbar decompression stated that they would not participate in a randomised placebo-controlled surgical trial due to concerns about costs to themselves [33].

#### Consent and information procedures

The consent and information procedures used also emerged as frequent themes influencing participation. Informed consent is an essential part of any intervention experiment, however variations in the presentation of this information impacted willingness to participate. Overall, as part of the information process, patients tend to want information about the potential risks and benefits of participating, and the perception of having received this information influences motivation to participate. In one survey of patients with spinal stenosis, when asked what would increase their willingness to participate in a placebo-controlled trial, 19.5% made responses around wanting reassurance of a good outcome [33]. Interviews of people with PD, who were enrolled or intending to enrol in a placebo surgery trial, found that most cited direct personal benefit as motivation for participating in the trial, with a proportion of these stating that their belief over their chance of benefit was based on information from the researcher or patient information consent form [43, 44].

In addition to patients’ preferences for more information on study benefits, several studies also suggested that a patient’s acceptance of placebo surgery trials is dependent on the provision of sufficient information and understanding overall [33, 39, 46]. Other authors also state that properly educating and informing patients, with a focus on the balance of risks, is particularly critical in RCTs [47, 48]. The need for comprehensive study information is also highlighted in studies that test patients understanding of critical aspects of trial design. One placebo trial found that whilst the majority of participants understood the rationale, purpose, random assignment, procedures and conditions under which they would be offered to crossover to the intervention arm if allocated to placebo surgery, 32% of participants assumed that crossover to active treatment would be offered without condition, or were unsure of the conditions [41]. Greater patient involvement in the consent process (such as writing consent in own hand, allowing thorough discussion, and use of formal decision aids) may also influence willingness to participate and increase the patient-centredness of the recruitment procedure [36]. Surgeon involvement in the information and consent procedures was also noted as a factor affecting willingness to participate. In one study, 69% of patients participating in an RCT of arthroscopic partial meniscectomy versus placebo surgery considered the oral information from the orthopaedic surgeon as the most important, compared to information presented in writing or on a DVD [40].

Another aspect of the information and consent processes purported to considerably influence patients’ attitudes towards placebo surgery trials is the terminology used, particularly the word ‘sham’, sometimes used to describe a placebo procedure. Campbell (2010) conducted a thorough investigation into patient attitudes and preferences for sham surgery in orthopaedics. Although they do not provide specific details regarding their discussions of terminology with patients, they state that the choice of word (sham or placebo) can lead to markedly different perceptions, and that placebo surgery tended to be a more acceptable descriptor [36].

### Critical appraisal of included studies

We used the MMAT [25] to assess qualitative, quantitative, and mixed method studies (Additional file 3: Appendix 3). The included descriptive quantitative studies (*n*=2) [34, 40] had methodological limitations, primarily high risk of non-response bias. The quality of the included qualitative and mixed methods papers was generally high (*n*=11) [12, 33, 36, 37, 39, 41–46]. The remaining publications were narrative reviews (*n*=4) [35, 38, 48, 49], or guidelines (*n*=1) [47]. The quality of these publications could not be assessed using the MMAT. We applied the approach described in the “Methods” section, whereby the quality of these papers was considered sufficient if they appeared in an international peer-reviewed journal. This approach to quality assessment has been used in other reviews [30, 31]. All five of these publications met this threshold and referenced other peer-reviewed literature. No further assessment of quality was made within this group. Studies were not excluded based on quality. The assessment of quality for each of the included studies is presented in the supplementary appendix.

## Discussion

This scoping review found that several themes, including the unique characteristics and views of patients and clinicians, as well as characteristics of the trial design itself, are likely to affect willingness to participate in randomised placebo-controlled surgical trials. Despite an increasing emphasis on the importance of RCTs in orthopaedic surgery [13], low recruitment is consistently identified as a threat to the validity of these trials [13]. Considering the preferences of trial participants, including both patients and clinicians, and their effects on decision-making, may improve trial design and recruitment, and ensure trials are both feasible and patient-centred.

In this review, we showed that several patient characteristics were associated with being more or less likely to agree to participate in a surgical placebo-controlled RCT. Some aspects, such as clinical characteristics (level of pain, disability, or disease state) are important to consider when recruiting to trials. If only patients with low levels of pain or disability are willing to participate, the generalisability of the trial data may be threatened. There are also ethical considerations around potential participants’ level of pain and the accessibility offered by a trial. Further, it is also important to consider that most people who enrolled in placebo surgery trials or indicated a willingness to do so, expressed a desire and/or belief that they would obtain some therapeutic benefit themselves. This finding has also been shown in studies of trial participation across other therapeutic areas including cancer [50] and may have implications for obtaining valid consent from patients. Allowing people to participate in a trial due to their own belief that they will benefit, poses an ethical dilemma, given that equipoise should be a prerequisite condition in any RCT [51]. Finally, altruism and a desire to benefit science also increased willingness to participate, which has been similarly reported across several other studies [40, 52].

Some of the results for patient characteristics weren’t consistent across studies, for example, education was differentially reported to affect recruitment. These may therefore not be consistent predictors of willingness to participate, or may reflect the pool of participants included in the study rather than factors influencing decisions to participate.

Although clinician endorsement is essential for the success of clinical trials, studies have consistently shown that a significant proportion of physicians are unwilling to participate [12, 37, 48]. The barriers to participation may vary, but include lack of time and resources, trial-specific issues, communication difficulties, conflicts between the role of clinician and scientist, and inadequate research experience and training for physicians. Here, we found that with respect to surgical placebo-controlled RCTs, surgeons and anaesthetists were concerned with the active deception required to participate in a randomised, blinded trial of surgery [12, 34, 48], despite not generally being opposed to the concept of placebo [12]. This may be due to the greater potential harms perceived to be associated with surgery compared with other areas of medicine such as anaesthesia, bleeding, infection, and additional pain [53]. It has been suggested that the physician who performs the trial procedures could be removed from providing postoperative care, in order to try and limit the need for the surgeons to participate in this deception [54], however as yet it is unclear whether this approach has been tested. Clinicians with more research training were more likely to agree to participate in placebo surgery trials. Although perceived conflict between roles as a scientist (conducting a placebo-controlled trial) and a clinician (caring for/treating patients) led to an increased likelihood of declining to participate, primarily due to concerns about deceiving patients [34, 48], greater prior experience with research methods or training in clinical trials was associated with an increased willingness to participate [34, 49]. It is possible that training in research and the scientific method may help overcome the conflict between being a scientist and a clinician which may be associated with a placebo-controlled surgery trial. It also appeared that for a surgeon to be willing to recruit patients to a trial and/or participate, the surgeon must believe there is a real question to be answered, in particular, that the true effectiveness of the procedure is unknown, commonly referred to as clinical equipoise [48]. Ensuring research education throughout medical training, fostering the development of inquiry and research questions that align with physician interests and have potential to improve patient care will help to develop clinician-researchers and potentially improve physician participation in trials, which may improve trial quality [55]. This aligns with a number of recent calls to increase the number of surgeon-scientists, as they have a unique ability to draw from clinical observation to inform the development of new therapeutic strategies [56, 57].

Broadly, several trial design features were consistently identified as impacting decisions to participate, which may inform future design of patient-centred trials. Addressing some of the issues commonly raised, particularly around patients’ needs for high-quality information and improving informed consent, will increase the quality and safety of trial participation and thereby assist in improving trial recruitment and retention. Our findings agree with other recent research, which suggests that the informed consent procedure for trials that involve placebo operations is frequently imperfect [35, 36, 47, 48]. A therapeutic misconception may exist for many patients, where an assumption is made that any intervention offered will have some therapeutic value. The prevalence of this phenomenon, which has been documented in other studies, undermines the idea that potential trial participants objectively and critically evaluate information presented to them around trial participation [44]. Taking into account patient preferences around trial design may improve the flow of information and contribute towards ensuring trials are more patient-centred. Until patient-centred trial methods are more frequently deployed, it is uncertain whether potential interventions to ensure patient-centredness will translate into improvements for patients and for trial fidelity. However, adopting more patient-centred approaches to the conduct of surgical RCTs, such as continuous participant engagement and feedback, and addressing the information needs of diverse populations is likely to improve trial recruitment and retention.

Clear and explicit communication regarding trial design at the outset may help patients understand some relatively unfamiliar concepts, such as randomisation, sham/placebo, likelihood of therapeutic benefit, and crossover. Although informed consent is a required component of trial design, in this review we found that many patients did not accurately understand these concepts when asked to recall them. There is a need to develop interventions which more effectively communicate these concepts to patients.

This scoping review identified several important implications for patient-centred trial design. The decision to participate in a surgical RCT, for both patients and clinicians, appears to be sensitive to values and preferences. Such attributes should be considered and incorporated into trial design where possible. Although not all identified attributes are manipulable (such as individual characteristics of patients or surgeons), and not all that are manipulable are ethical to manipulate (such as assurance of a good clinical outcome in terms of pain reduction), there are aspects of trial design that can be adapted to ensure optimal recruitment while still adhering to best scientific practice. The factors from this scoping review which have potential to be adjusted include the terminology used to define the sham/placebo element, the invasiveness of the placebo procedure, the randomisation ratio and presence of other arms, the involvement of the clinician/surgeon in the trial, whether crossover is offered as part of the trial and when, and potentially depending on funding bodies, the financial costs of the placebo procedure.

Although much has been published about patient- and physician-centred trials, with preferences frequently identified as a threat to the validity of RCTs, there have been very few quantitative approaches used to estimate the effects on recruitment and retention. One important next step is to experimentally determine the influence that these factors have on willingness to participate in placebo surgery trials. This list of potentially manipulable factors will provide an important contribution to the development of attributes for a DCE providing information about patient and surgeon willingness to participate in placebo surgery trials and balance trade-offs among specific features of trials. This experiment will assist in future trial design based on patient and surgeon preferences and generate improved economic information about conducting trials with varying recruitment rates. Further, investigating conflicts between surgeon and patient preferences, such as the preferred level of surgeon involvement in a trial, may also be important in terms of recruitment. If the surgeon and patient preferences are in direct contrast, this creates a dilemma in designing a trial aimed at improving recruitment.

In this review, we have characterised several potentially relevant attributes; however, there is still a risk that none of the preferred characteristics will be salient enough to override the unwillingness to participate in a placebo or sham surgery trial, particularly given perceived risks and time requirements. There is also a risk that there are other important factors which have not yet been captured by the exiting literature. Further qualitative research is needed to ensure the full range of preferences for placebo surgery trials are understood. A study conducted by members of this research team, interviewing patients and surgeons about their understanding of and preferences for placebo surgery trials [58] will contribute to this need for future research and help to inform the DCE.

Despite the fact that our search strategy was designed to identify existing quantitative preference studies, we didn’t find any existing preference-based study designs, such as the rating scale, visual analogue scale, standard gamble, time trade-off, contingent valuation, DCE, and best-worst scaling. One study included an item in the questionnaire that resembled a DCE in that it asked respondents to consider two study design options within a clinical scenario, indicate their preferred option and their willingness to participate in that study [34]. Although the format of the question resembled a choice task, it was only a single question without the methodological rigour of a full choice experiment. Future studies should increase the focus on quantifying preferences and their effects on decision-making and generalisability in the context of clinical trials in surgery to continue to improve the trial design. Quantifying preferences for surgical trial design, in addition to describing them, is important to inform the development of patient and clinician centred trials by identifying the variables most likely to impact decisions to participate. It is likely that by taking preferences into account in trial design, recruitment to trials will be improved. In obtaining a quantifiable outcome as to the extent that each factor influences participation, these outcomes can be readily incorporated into economic and feasibility models to provide more concrete estimates of the level of recruitment for a particular trial design.

## Limitations

We identified a heterogeneous group of publications, including reviews, interviews, and surveys. Some of the studies were relatively poorly described, referred to a very specific group of patients in a single setting, or had a high risk of bias, which may limit the generalisability of findings. Further, we only included English language publications. Nevertheless, we were able to identify several common themes and patterns among the identified studies, which we believe are broadly applicable to surgical trials.

## Conclusion

Participant recruitment is a significant challenge in placebo surgery trials. Low recruitment may reduce the conclusions we can make based on a study, introduce bias, threaten external validity, and increase the risk of trial abandonment. Failing to complete a trial or completing a trial with a reduced number of patients compromises knowledge gains and exposes patients to the risks of surgery without justification, all of which are significant scientific and ethical concerns. It is important to enhance our understanding of the role patient and clinician preferences may play in surgical trials, and in particular a quantification of these is needed to optimise the design and implementation of RCTs in surgery.

## Supplementary Information

**Additional file 1: Appendix 1.** PRISMA-ScR

**Additional file 2: Appendix 2.** Search strategy

**Additional file 3: Appendix 3.** Data extraction sheet

**Additional file 4: Appendix 4.** MMAT criteria

**Additional file 5: Appendix 5.** MMAT assessment

## Data Availability

The datasets used and/or analysed during the current study are available from the corresponding author on reasonable request.
