# Supplementary material for: Patient and clinician characteristics and preferences for increasing participation in placebo surgery trials: a scoping review of attributes to inform a discrete choice experiment
Source: Trials. 2022 Apr 12;23:296. doi: 10.1186/s13063-022-06277-x (PMC9006556; doi:10.1186/s13063-022-06277-x)
Supplement: Supplementary file 1 — Additional file 1: Appendix 1. PRISMA-ScR [file 13063_2022_6277_MOESM1_ESM.docx]

**Preferred Reporting Items for Systematic reviews and Meta-Analyses extension for Scoping Reviews (PRISMA-ScR) Checklist**

| **SECTION** | **ITEM** | **PRISMA-ScR CHECKLIST ITEM** | **REPORTED ON PAGE #** |
| --- | --- | --- | --- |
| **TITLE** | | | |
| Title | 1 | Identify the report as a scoping review. | 1 |
| **ABSTRACT** | | | |
| Structured summary | 2 | Provide a structured summary that includes (as applicable): background, objectives, eligibility criteria, sources of evidence, charting methods, results, and conclusions that relate to the review questions and objectives. | 3 |
| **INTRODUCTION** | | | |
| Rationale | 3 | Describe the rationale for the review in the context of what is already known. Explain why the review questions/objectives lend themselves to a scoping review approach. | 4 |
| Objectives | 4 | Provide an explicit statement of the questions and objectives being addressed with reference to their key elements (e.g., population or participants, concepts, and context) or other relevant key elements used to conceptualize the review questions and/or objectives. | 4 |
| **METHODS** | | | |
| Protocol and registration | 5 | Indicate whether a review protocol exists; state if and where it can be accessed (e.g., a Web address); and if available, provide registration information, including the registration number. | 5 |
| Eligibility criteria | 6 | Specify characteristics of the sources of evidence used as eligibility criteria (e.g., years considered, language, and publication status), and provide a rationale. | 5-6 |
| Information sources* | 7 | Describe all information sources in the search (e.g., databases with dates of coverage and contact with authors to identify additional sources), as well as the date the most recent search was executed. | 5 |
| Search | 8 | Present the full electronic search strategy for at least 1 database, including any limits used, such that it could be repeated. | Appendix 2 |
| Selection of sources of evidence† | 9 | State the process for selecting sources of evidence (i.e., screening and eligibility) included in the scoping review. | 5-6 |
| Data charting process‡ | 10 | Describe the methods of charting data from the included sources of evidence (e.g., calibrated forms or forms that have been tested by the team before their use, and whether data charting was done independently or in duplicate) and any processes for obtaining and confirming data from investigators. | 6 |
| Data items | 11 | List and define all variables for which data were sought and any assumptions and simplifications made. | 6 |
| Critical appraisal of individual sources of evidence§ | 12 | If done, provide a rationale for conducting a critical appraisal of included sources of evidence; describe the methods used and how this information was used in any data synthesis (if appropriate). | 6 |
| Synthesis of results | 13 | Describe the methods of handling and summarizing the data that were charted. | 6 |
| **RESULTS** | | | |
| Selection of sources of evidence | 14 | Give numbers of sources of evidence screened, assessed for eligibility, and included in the review, with reasons for exclusions at each stage, ideally using a flow diagram. | 7 |
| Characteristics of sources of evidence | 15 | For each source of evidence, present characteristics for which data were charted and provide the citations. | 7-8 |
| Critical appraisal within sources of evidence | 16 | If done, present data on critical appraisal of included sources of evidence (see item 12). | 15 |
| Results of individual sources of evidence | 17 | For each included source of evidence, present the relevant data that were charted that relate to the review questions and objectives. | Tables 2-4 |
| Synthesis of results | 18 | Summarize and/or present the charting results as they relate to the review questions and objectives. | 9-15 |
| **DISCUSSION** | | | |
| Summary of evidence | 19 | Summarize the main results (including an overview of concepts, themes, and types of evidence available), link to the review questions and objectives, and consider the relevance to key groups. | 15-17 |
| Limitations | 20 | Discuss the limitations of the scoping review process. | 17 |
| Conclusions | 21 | Provide a general interpretation of the results with respect to the review questions and objectives, as well as potential implications and/or next steps. | 17 |
| **FUNDING** | | | |
| Funding | 22 | Describe sources of funding for the included sources of evidence, as well as sources of funding for the scoping review. Describe the role of the funders of the scoping review. | 1 |

JBI = Joanna Briggs Institute; PRISMA-ScR = Preferred Reporting Items for Systematic reviews and Meta-Analyses extension for Scoping Reviews.

* Where *sources of evidence* (see second footnote) are compiled from, such as bibliographic databases, social media platforms, and Web sites.

† A more inclusive/heterogeneous term used to account for the different types of evidence or data sources (e.g., quantitative and/or qualitative research, expert opinion, and policy documents) that may be eligible in a scoping review as opposed to only studies. This is not to be confused with *information sources* (see first footnote).

‡ The frameworks by Arksey and O’Malley (6) and Levac and colleagues (7) and the JBI guidance (4, 5) refer to the process of data extraction in a scoping review as data charting*.*

§ The process of systematically examining research evidence to assess its validity, results, and relevance before using it to inform a decision. This term is used for items 12 and 19 instead of "risk of bias" (which is more applicable to systematic reviews of interventions) to include and acknowledge the various sources of evidence that may be used in a scoping review (e.g., quantitative and/or qualitative research, expert opinion, and policy document).

*From:* Tricco AC, Lillie E, Zarin W, O'Brien KK, Colquhoun H, Levac D, et al. PRISMA Extension for Scoping Reviews (PRISMAScR): Checklist and Explanation. Ann Intern Med. 2018;169:467–473.

**Search strategy: MEDLINE, Embase, and PsycINFO**

Database: Ovid MEDLINE(R) and Epub Ahead of Print, In-Process & Other Non-Indexed Citations and Daily <1946 to September 04, 2020>

Search Strategy:

--------------------------------------------------------------------------------

1 Clinical trials as topic/ or Controlled-clinical trials as topic/ (198156)

2 Randomized controlled trials as topic/ (135889)

3 Research design/ (108258)

4 Feasibility studies/ (68691)

5 1 or 2 or 3 or 4 (483418)

6 Placebo effect/ or Placebos/ (38966)

7 5 and 6 (14375)

8 exp Orthopedic procedures/ (307752)

9 exp Surgical procedures, Operative/ (3152513)

10 General surgery/ (38913)

11 Surgery.fs. (1990028)

12 exp Endoscopy/ (353546)

13 8 or 9 or 10 or 11 or 12 (3795498)

14 7 and 13 (1165)

15 (sham or imitation or dummy or placebo).ti,ab,kw,kf. (308651)

16 (placebo adj (control* or trial*)).ti,ab,kw,kf. (91130)

17 (surg* or arthroscop* or endoscop* or laparoscop*).ti,ab,kw. (2139014)

18 (15 or 16) and 17 (33481)

19 14 or 18 (34342)

20 Decision making/ (95666)

21 (decision* adj1 (make or made or making)).ti,ab,kw,kf. (151101)

22 Choice behavior/ or Consumer behavior/ or exp Motivation/ or choice behavio?r.ti,ab,kw. (219978)

23 "Patient acceptance of health care"/ (47249)

24 Refusal to participate/ (619)

25 exp Patient satisfaction/ or Patient selection/ or Patient participation/ (176559)

26 (patient* adj5 (prefer* or participat* or perspective* or choice* or attitude* or expectation* or willing* or accept* or view* or opinion* or belief*)).ti,ab,kw,kf. (187549)

27 Research subjects/px (1156)

28 "Attitude of Health Personnel"/ or Attitude to Health/ or "Health knowledge, attitudes and practice"/ (198120)

29 Clinical Decision-Making/ or exp Decision support techniques/ (86179)

30 Practice Patterns, Physicians'/ (60253)

31 ((physician* or surgeon* or clinician* or doctor* or health professional* or health practitioner* or stakeholder*) adj5 (prefer* or participat* or perspective* or choice* or attitude* or expectation* or willing* or view* or opinion* or belief*)).ti,ab,kw,kf. (60696)

32 20 or 21 or 22 or 23 or 24 or 25 or 26 or 27 or 28 or 29 or 30 or 31 (1055768)

33 19 and 32 (1398)

Annotation: Sham Surg AND Patient particip

34 exp animals/ not humans.sh. (4731219)

35 33 not 34 (1175)

36 limit 35 to (comment or editorial or letter or news) (19)

37 35 not 36 (1156)

38 limit 37 to english language (1098)

39 limit 38 to ed=20191024-20200907 (40)

Database: Embase <1947 to present>

Search Strategy:

--------------------------------------------------------------------------------

1 sham procedure/ (12042)

2 exp surgery/ (5330302)

3 exp endoscopy/ (656911)

4 surgery.fs. (2179364)

5 2 or 3 or 4 (6041032)

6 1 and 5 (5005)

7 (sham or imitation or dummy or placebo).ti,ab,kw. (445113)

8 (placebo adj (control* or trial*)).ti,ab,kw. (130125)

9 (surg* or arthroscop* or endoscop* or laparoscop*).ti,ab,kw. (3110466)

10 (7 or 8) and 9 (49855)

11 6 or 10 (53004)

12 decision making/ (235856)

13 (decision* adj1 (make or made or making)).ti,ab,kw. (206934)

14 consumer attitude/ (4905)

15 motivation/ (109050)

16 choice behavio?r.ti,ab,kw. (1839)

17 exp patient attitude/ or patient selection/ (495434)

18 (patient* adj5 (prefer* or participat* or perspective* or choice* or attitude* or expectation* or willing* or accept* or view* or opinion* or belief*)).ti,ab,kw. (296654)

19 research subject/ (7620)

20 health personnel attitude/ (83028)

21 physician attitude/ (53939)

22 attitude to health/ (115062)

23 clinical decision making/ (46828)

24 decision support system/ (22498)

25 clinical practice/ (290432)

26 ((physician* or surgeon* or clinician* or doctor* or health professional* or health practitioner* or stakeholder*) adj5 (prefer* or participat* or perspective* or choice* or attitude* or expectation* or willing* or view* or opinion* or belief*)).ti,ab,kw. (84737)

27 12 or 13 or 14 or 15 or 16 or 17 or 18 or 19 or 20 or 21 or 22 or 23 or 24 or 25 or 26 (1656408)

28 11 and 27 (2709)

29 (animal/ or nonhuman/) not human/ (6149848)

30 28 not 29 (2605)

31 limit 30 to (books or chapter or conference abstract or conference paper or "conference review" or editorial or letter or note) (728)

32 30 not 31 (1877)

33 limit 32 to english language (1802)

34 limit 33 to dd=20191024-20200907 (20)

Database: APA PsycInfo <1806 to August Week 5 2020>

Search Strategy:

--------------------------------------------------------------------------------

1 exp clinical trials/ (12392)

2 placebo/ (5699)

3 1 and 2 (925)

4 exp surgery/ (71881)

5 3 and 4 (19)

6 (sham or imitation or dummy or placebo).ti,ab,id. (61873)

7 (placebo adj (control* or trial*)).ti,ab,id. (16318)

8 (surg* or arthroscop* or endoscop* or laparoscop*).ti,ab,id. (50071)

9 (6 or 7) and 8 (2286)

10 5 or 9 (2295)

11 decision making/ or exp choice behavior/ (114946)

12 (decision* adj1 (make or made or making)).ti,ab,id. (106273)

13 exp motivation/ (141507)

14 choice behavio?r.ti,ab,id. (4783)

15 exp client attitudes/ (22306)

16 client participation/ or expectations/ (22306)

17 (patient* adj5 (prefer* or participat* or perspective* or choice* or attitude* or expectation* or willing* or accept* or view* or opinion* or belief*)).ti,ab,id. (44807)

18 experimental subjects/ or experimental recruitment/ or ethics/ (19319)

19 health personnel attitudes/ (19686)

20 health attitudes/ (10320)

21 ((physician* or surgeon* or clinician* or doctor* or health professional* or health practitioner* or stakeholder*) adj5 (prefer* or participat* or perspective* or choice* or attitude* or expectation* or willing* or view* or opinion* or belief*)).ti,ab,id. (19158)

22 11 or 12 or 13 or 14 or 15 or 16 or 17 or 18 or 19 or 20 or 21 (415237)

23 10 and 22 (77)

24 limit 23 to human (47)

25 limit 24 to up=20191024-20200907 (1)

**Quality appraisal: Mixed methods appraisal tool (MMAT)**

**Quality assessment of studies using MMAT**

| **Study** | **SCREENING QUESTIONS** | | **QUALITATIVE STUDIES** | | | | | **4. QUANTITATIVE DESCRIPTIVE STUDIES** | | | | | **5. MIXED METHODS STUDIES** | | | | |
| --- | --- | --- | --- | --- | --- | --- | --- | --- | --- | --- | --- | --- | --- | --- | --- | --- | --- |
|  | **(1)** | **(2)** | **(1)** | **(2)** | **(3)** | **(4)** | **(5)** | **(1)** | **(2)** | **(3)** | **(4)** | **(5)** | **(1)** | **(2)** | **(3)** | **(4)** | **(5)** |
| Anderson 2019 | ✓ | ✓ |  |  |  |  |  | ✓ | ✓ | ✓ | ✓ | ✓ |  |  |  |  |  |
| Baldwin 2016 | ✓ | ✓ |  |  |  |  |  | ✓ | ✓ | ✓ | 🗶 | ✓ |  |  |  |  |  |
| Campbell 2010a | ✓ | ✓ |  |  |  |  |  |  |  |  |  |  | ✓ | ✓ | ✓ | ✓ | ✓ |
| Campbell 2010b | - | - | - | - | ✓ | ✓ | ✓ |  |  |  |  |  |  |  |  |  |  |
| Campbell 2011 | ✓ | ✓ | ✓ | ✓ | ✓ | ✓ | ✓ |  |  |  |  |  |  |  |  |  |  |
| Frank 2008 | ✓ | ✓ | ✓ | ✓ | ✓ | ✓ | ✓ |  |  |  |  |  |  |  |  |  |  |
| Hare 2014 | ✓ | ✓ |  |  |  |  |  | ✓ | - | ✓ | 🗶 | ✓ |  |  |  |  |  |
| Kim 2012a | ✓ | ✓ | ✓ | ✓ | ✓ | ✓ | ✓ |  |  |  |  |  |  |  |  |  |  |
| Kim 2012b | ✓ | ✓ | ✓ | ✓ | ✓ | ✓ | ✓ |  |  |  |  |  |  |  |  |  |  |
| Kim 2013 | ✓ | ✓ | ✓ | ✓ | - | ✓ | ✓ |  |  |  |  |  |  |  |  |  |  |
| Kim 2015 | ✓ | ✓ | ✓ | ✓ | ✓ | ✓ | ✓ |  |  |  |  |  |  |  |  |  |  |
| Rios 2021 | ✓ | ✓ | ✓ | ✓ | ✓ | ✓ | ✓ |  |  |  |  |  |  |  |  |  |  |
| Swift 2012 | ✓ | ✓ | ✓ | ✓ | ✓ | ✓ | ✓ |  |  |  |  |  |  |  |  |  |  |
| Wartolowska 2014 | ✓ | ✓ | ✓ | ✓ | ✓ | ✓ | ✓ |  |  |  |  |  |  |  |  |  |  |

✓ Denotes criterion met; 🗶 Denotes criterion not met; - Denotes insufficient information provided to determine if criterion met (MMAT response ‘Can’t tell’)

Note: No randomised or non-randomised studies were included and these aspects of the MMAT have not been included in the table.

Screening criteria: (1) Are there clear research questions?; (2) Do the collected data allow to address the research questions?

Qualitative criteria: (1) Is the qualitative approach appropriate to answer the research question?; (2) Are the qualitative data collection methods adequate to address the research question?; (3) Are the findings adequately derived from the data?; (4) Is the interpretation of results sufficiently substantiated by data?; (5) Is there coherence between qualitative data sources, collection, analysis and interpretation?

Quantitative descriptive criteria: (1) Is the sampling strategy relevant to address the research question?; (2) Is the sample representative of the target population?; (3) Are the measurements appropriate?; (4) Is the risk of nonresponse bias low?; (5) Is the statistical analysis appropriate to answer the research question?

Mixed methods criteria: (1) Is there an adequate rationale for using a mixed methods design to address the research question?; (2) Are the different components of the study effectively integrated to answer the research question?; (3) Are the outputs of the integration of qualitative and quantitative components adequately interpreted?; (4) Are divergences and inconsistencies between quantitative and qualitative results adequately addressed?; (5) Do the different components of the study adhere to the quality criteria of each tradition of the methods involved?
