# Supplementary material for: Patient and clinician characteristics and preferences for increasing participation in placebo surgery trials: a scoping review of attributes to inform a discrete choice experiment
Source: Trials. 2022 Apr 12;23:296. doi: 10.1186/s13063-022-06277-x (PMC9006556; doi:10.1186/s13063-022-06277-x)
Supplement: Supplementary file 2 — Additional file 2: Appendix 2. Search strategy [file 13063_2022_6277_MOESM2_ESM.doc]

Database: Ovid MEDLINE(R) and Epub Ahead of Print, In-Process & Other Non-Indexed Citations and Daily <1946 to September 04, 2020>

Search Strategy:

--------------------------------------------------------------------------------

1 Clinical trials as topic/ or Controlled-clinical trials as topic/ (198156)

2 Randomized controlled trials as topic/ (135889)

3 Research design/ (108258)

4 Feasibility studies/ (68691)

5 1 or 2 or 3 or 4 (483418)

6 Placebo effect/ or Placebos/ (38966)

7 5 and 6 (14375)

8 exp Orthopedic procedures/ (307752)

9 exp Surgical procedures, Operative/ (3152513)

10 General surgery/ (38913)

11 Surgery.fs. (1990028)

12 exp Endoscopy/ (353546)

13 8 or 9 or 10 or 11 or 12 (3795498)

14 7 and 13 (1165)

15 (sham or imitation or dummy or placebo).ti,ab,kw,kf. (308651)

16 (placebo adj (control* or trial*)).ti,ab,kw,kf. (91130)

17 (surg* or arthroscop* or endoscop* or laparoscop*).ti,ab,kw. (2139014)

18 (15 or 16) and 17 (33481)

19 14 or 18 (34342)

20 Decision making/ (95666)

21 (decision* adj1 (make or made or making)).ti,ab,kw,kf. (151101)

22 Choice behavior/ or Consumer behavior/ or exp Motivation/ or choice behavio?r.ti,ab,kw. (219978)

23 "Patient acceptance of health care"/ (47249)

24 Refusal to participate/ (619)

25 exp Patient satisfaction/ or Patient selection/ or Patient participation/ (176559)

26 (patient* adj5 (prefer* or participat* or perspective* or choice* or attitude* or expectation* or willing* or accept* or view* or opinion* or belief*)).ti,ab,kw,kf. (187549)

27 Research subjects/px (1156)

28 "Attitude of Health Personnel"/ or Attitude to Health/ or "Health knowledge, attitudes and practice"/ (198120)

29 Clinical Decision-Making/ or exp Decision support techniques/ (86179)

30 Practice Patterns, Physicians'/ (60253)

31 ((physician* or surgeon* or clinician* or doctor* or health professional* or health practitioner* or stakeholder*) adj5 (prefer* or participat* or perspective* or choice* or attitude* or expectation* or willing* or view* or opinion* or belief*)).ti,ab,kw,kf. (60696)

32 20 or 21 or 22 or 23 or 24 or 25 or 26 or 27 or 28 or 29 or 30 or 31 (1055768)

33 19 and 32 (1398)

Annotation: Sham Surg AND Patient particip

34 exp animals/ not humans.sh. (4731219)

35 33 not 34 (1175)

36 limit 35 to (comment or editorial or letter or news) (19)

37 35 not 36 (1156)

38 limit 37 to english language (1098)

39 limit 38 to ed=20191024-20200907 (40)

Database: Embase <1947 to present>

Search Strategy:

--------------------------------------------------------------------------------

1 sham procedure/ (12042)

2 exp surgery/ (5330302)

3 exp endoscopy/ (656911)

4 surgery.fs. (2179364)

5 2 or 3 or 4 (6041032)

6 1 and 5 (5005)

7 (sham or imitation or dummy or placebo).ti,ab,kw. (445113)

8 (placebo adj (control* or trial*)).ti,ab,kw. (130125)

9 (surg* or arthroscop* or endoscop* or laparoscop*).ti,ab,kw. (3110466)

10 (7 or 8) and 9 (49855)

11 6 or 10 (53004)

12 decision making/ (235856)

13 (decision* adj1 (make or made or making)).ti,ab,kw. (206934)

14 consumer attitude/ (4905)

15 motivation/ (109050)

16 choice behavio?r.ti,ab,kw. (1839)

17 exp patient attitude/ or patient selection/ (495434)

18 (patient* adj5 (prefer* or participat* or perspective* or choice* or attitude* or expectation* or willing* or accept* or view* or opinion* or belief*)).ti,ab,kw. (296654)

19 research subject/ (7620)

20 health personnel attitude/ (83028)

21 physician attitude/ (53939)

22 attitude to health/ (115062)

23 clinical decision making/ (46828)

24 decision support system/ (22498)

25 clinical practice/ (290432)

26 ((physician* or surgeon* or clinician* or doctor* or health professional* or health practitioner* or stakeholder*) adj5 (prefer* or participat* or perspective* or choice* or attitude* or expectation* or willing* or view* or opinion* or belief*)).ti,ab,kw. (84737)

27 12 or 13 or 14 or 15 or 16 or 17 or 18 or 19 or 20 or 21 or 22 or 23 or 24 or 25 or 26 (1656408)

28 11 and 27 (2709)

29 (animal/ or nonhuman/) not human/ (6149848)

30 28 not 29 (2605)

31 limit 30 to (books or chapter or conference abstract or conference paper or "conference review" or editorial or letter or note) (728)

32 30 not 31 (1877)

33 limit 32 to english language (1802)

34 limit 33 to dd=20191024-20200907 (20)

Database: APA PsycInfo <1806 to August Week 5 2020>

Search Strategy:

--------------------------------------------------------------------------------

1 exp clinical trials/ (12392)

2 placebo/ (5699)

3 1 and 2 (925)

4 exp surgery/ (71881)

5 3 and 4 (19)

6 (sham or imitation or dummy or placebo).ti,ab,id. (61873)

7 (placebo adj (control* or trial*)).ti,ab,id. (16318)

8 (surg* or arthroscop* or endoscop* or laparoscop*).ti,ab,id. (50071)

9 (6 or 7) and 8 (2286)

10 5 or 9 (2295)

11 decision making/ or exp choice behavior/ (114946)

12 (decision* adj1 (make or made or making)).ti,ab,id. (106273)

13 exp motivation/ (141507)

14 choice behavio?r.ti,ab,id. (4783)

15 exp client attitudes/ (22306)

16 client participation/ or expectations/ (22306)

17 (patient* adj5 (prefer* or participat* or perspective* or choice* or attitude* or expectation* or willing* or accept* or view* or opinion* or belief*)).ti,ab,id. (44807)

18 experimental subjects/ or experimental recruitment/ or ethics/ (19319)

19 health personnel attitudes/ (19686)

20 health attitudes/ (10320)

21 ((physician* or surgeon* or clinician* or doctor* or health professional* or health practitioner* or stakeholder*) adj5 (prefer* or participat* or perspective* or choice* or attitude* or expectation* or willing* or view* or opinion* or belief*)).ti,ab,id. (19158)

22 11 or 12 or 13 or 14 or 15 or 16 or 17 or 18 or 19 or 20 or 21 (415237)

23 10 and 22 (77)

24 limit 23 to human (47)

25 limit 24 to up=20191024-20200907 (1)
