# Supplementary material for: Patient and clinician characteristics and preferences for increasing participation in placebo surgery trials: a scoping review of attributes to inform a discrete choice experiment
Source: Trials. 2022 Apr 12;23:296. doi: 10.1186/s13063-022-06277-x (PMC9006556; doi:10.1186/s13063-022-06277-x)
Supplement: Supplementary file 3 — Additional file 3: Appendix 3. Data extraction sheet [file 13063_2022_6277_MOESM3_ESM.doc]

**Appendix 3**

**Data Extraction Sheet: Exploring themes associated with recruitment to placebo controlled trials in surgery**

| Reviewer initials | Article | Year | Country | Surgical specialty | Study method or design | Dataset | Overall participation/  recruitment rate | Patient characteristics | Surgeon characteristics | Trial characteristics | Recruitment/consent/ information factors | Notes/context |
| --- | --- | --- | --- | --- | --- | --- | --- | --- | --- | --- | --- | --- |
| MH, LW, DL | Author and date |  |  | e.g. orthopaedics, neurosurgery | Interview, DCE, review, etc | No. and type of patients or clinicians | (if reported) |  |  |  |  |  |
|  |  |  |  |  |  |  |  |  |  |  |  |  |
|  |  |  |  |  |  |  |  |  |  |  |  |  |
|  |  |  |  |  |  |  |  |  |  |  |  |  |
