# Supplementary material for: Patient and clinician characteristics and preferences for increasing participation in placebo surgery trials: a scoping review of attributes to inform a discrete choice experiment
Source: Trials. 2022 Apr 12;23:296. doi: 10.1186/s13063-022-06277-x (PMC9006556; doi:10.1186/s13063-022-06277-x)
Supplement: Supplementary file 5 — Additional file 5: Appendix 5. MMAT assessment [file 13063_2022_6277_MOESM5_ESM.docx]

**Table 5: Quality assessment of studies using MMAT**

| **Study** | **SCREENING QUESTIONS** | | **QUALITATIVE STUDIES** | | | | | **4. QUANTITATIVE DESCRIPTIVE STUDIES** | | | | | **5. MIXED METHODS STUDIES** | | | | |
| --- | --- | --- | --- | --- | --- | --- | --- | --- | --- | --- | --- | --- | --- | --- | --- | --- | --- |
|  | **(1)** | **(2)** | **(1)** | **(2)** | **(3)** | **(4)** | **(5)** | **(1)** | **(2)** | **(3)** | **(4)** | **(5)** | **(1)** | **(2)** | **(3)** | **(4)** | **(5)** |
| Anderson 2019 | ✓ | ✓ |  |  |  |  |  | ✓ | ✓ | ✓ | ✓ | ✓ |  |  |  |  |  |
| Baldwin 2016 | ✓ | ✓ |  |  |  |  |  | ✓ | ✓ | ✓ | 🗶 | ✓ |  |  |  |  |  |
| Campbell 2010a | ✓ | ✓ |  |  |  |  |  |  |  |  |  |  | ✓ | ✓ | ✓ | ✓ | ✓ |
| Campbell 2010b | - | - | - | - | ✓ | ✓ | ✓ |  |  |  |  |  |  |  |  |  |  |
| Campbell 2011 | ✓ | ✓ | ✓ | ✓ | ✓ | ✓ | ✓ |  |  |  |  |  |  |  |  |  |  |
| Frank 2008 | ✓ | ✓ | ✓ | ✓ | ✓ | ✓ | ✓ |  |  |  |  |  |  |  |  |  |  |
| Hare 2014 | ✓ | ✓ |  |  |  |  |  | ✓ | - | ✓ | 🗶 | ✓ |  |  |  |  |  |
| Kim 2012a | ✓ | ✓ | ✓ | ✓ | ✓ | ✓ | ✓ |  |  |  |  |  |  |  |  |  |  |
| Kim 2012b | ✓ | ✓ | ✓ | ✓ | ✓ | ✓ | ✓ |  |  |  |  |  |  |  |  |  |  |
| Kim 2013 | ✓ | ✓ | ✓ | ✓ | - | ✓ | ✓ |  |  |  |  |  |  |  |  |  |  |
| Kim 2015 | ✓ | ✓ | ✓ | ✓ | ✓ | ✓ | ✓ |  |  |  |  |  |  |  |  |  |  |
| Rios 2021 | ✓ | ✓ | ✓ | ✓ | ✓ | ✓ | ✓ |  |  |  |  |  |  |  |  |  |  |
| Swift 2012 | ✓ | ✓ | ✓ | ✓ | ✓ | ✓ | ✓ |  |  |  |  |  |  |  |  |  |  |
| Wartolowska 2014 | ✓ | ✓ | ✓ | ✓ | ✓ | ✓ | ✓ |  |  |  |  |  |  |  |  |  |  |

✓ Denotes criterion met; 🗶 Denotes criterion not met; - Denotes insufficient information provided to determine if criterion met (MMAT response ‘Can’t tell’)

Note: No randomised or non-randomised studies were included and these aspects of the MMAT have not been included in the table.

Screening criteria: (1) Are there clear research questions?; (2) Do the collected data allow to address the research questions?

Qualitative criteria: (1) Is the qualitative approach appropriate to answer the research question?; (2) Are the qualitative data collection methods adequate to address the research question?; (3) Are the findings adequately derived from the data?; (4) Is the interpretation of results sufficiently substantiated by data?; (5) Is there coherence between qualitative data sources, collection, analysis and interpretation?

Quantitative descriptive criteria: (1) Is the sampling strategy relevant to address the research question?; (2) Is the sample representative of the target population?; (3) Are the measurements appropriate?; (4) Is the risk of nonresponse bias low?; (5) Is the statistical analysis appropriate to answer the research question?

Mixed methods criteria: (1) Is there an adequate rationale for using a mixed methods design to address the research question?; (2) Are the different components of the study effectively integrated to answer the research question?; (3) Are the outputs of the integration of qualitative and quantitative components adequately interpreted?; (4) Are divergences and inconsistencies between quantitative and qualitative results adequately addressed?; (5) Do the different components of the study adhere to the quality criteria of each tradition of the methods involved?
